# Validation of the nausea and vomiting of pregnancy specific health related quality of life questionnaire

**DOI:** 10.1186/1477-7525-6-32

**Published:** 2008-05-09

**Authors:** Anaïs Lacasse, Anick Bérard

**Affiliations:** 1 Faculty of Pharmacy, University of Montreal, Montreal, Quebec, Canada; 2 Research Center, CHU Sainte-Justine, Montreal, Quebec, Canada

## Abstract

**Background:**

The only existing NVP-specific quality of life (QOL) questionnaire is the "Health-Related Quality of Life for Nausea and Vomiting during Pregnancy" (NVPQOL). However, the reliability and validity of the NVPQOL have never been established. In order to justify its usage, the internal consistency and criterion validity of the NVPQOL questionnaire must be ascertained.

**Methods:**

A prospective observational study including pregnant women attending CHU Sainte-Justine or René-Laennec clinics for their prenatal care was conducted from 2004 to 2006. Women were eligible if they were ≥ 18 years of age and ≤ 16 weeks of gestation at the time of their first prenatal visit. During this initial visit, women who reported NVP were also asked to complete the NVPQOL and the SF-12. Cronbach's alpha coefficients were calculated as the measures of the internal consistency of the NVPQOL. With respect to the criterion validity, linear regression models were built to measure the association between the NVPQOL and the SF-12 scores.

**Results:**

Of the 367 women included in the study, 288 (78.5%) reported NVP in the first trimester of pregnancy. Among these women, the Cronbach's alpha coefficients were high for the complete NVPQOL questionnaire (α = 0.98), and for the four distinct domains [physical symptoms and aggravating factors (α = 0.90); fatigue (α = 0.94); emotions (α = 0.86); limitations (α = 0.97)]. NVP-specific QOL as measured by the NVPQOL was significantly associated with physical and mental QOL as measured by the SF-12.

**Conclusion:**

Our data suggest that the NVPQOL is a reliable and valid index to measure NVP-specific QOL in the first trimester of pregnancy.

## Background

Most pregnant women (50 to 90%) experience nausea and vomiting of pregnancy (NVP) during the first trimester [1]. NVP appears normally between the 4^th ^and 6^th ^week of gestation and peaks between week 8 and 12 [2,3]. Most of the symptoms disappear by the 20^th ^week of gestation [2]. A more severe form of NVP, called *hyperemesis gravidarum *(HG), can also occur in 0.5 to 3% of pregnancies [4,5], and has been found to be the most common reason for hospitalisation during the first trimester of pregnancy [6].

NVP can have a significant impact on family life, on the ability to perform usual daily activities, on social functioning [7], and on stress levels [8]. In addition, the presence and severity of NVP have been shown to have an impact on the quality of life (QOL) of pregnant women [9-11]. Since health-related QOL is a non-negligible outcome when evaluating the burden of illness of health problems, it is important to have a valid way of measuring this health issue. Some generic measures of health-related QOL are available, but the only existing NVP-specific QOL questionnaire is the "Health-Related Quality of Life for Nausea and Vomiting during Pregnancy" (NVPQOL) [12]. However, the reliability and criterion validity of the NVPQOL have never been established.

To improve confidence in using the NVPQOL, we aimed to measure the internal consistency of the NVPQOL questionnaire. In addition, we sought to establish the criterion validity of the NVPQOL questionnaire by measuring the extent to which it is associated with the generic QOL-SF-12.

## Methods

A prospective observational study on pregnant women having prenatal care at the obstetrics and gynaecology clinic of either the *Centre Hospitalier Universitaire Sainte-Justine *(CHU Sainte-Justine) or the René-Laennec clinic, both affiliated with the University of Montreal, Quebec, Canada was conducted from October 2004 to March 2006. Women were eligible if they were: 1) at least 18 years of age; 2) at their first prenatal visit at the obstetric and gynaecology clinic of the CHU Sainte-Justine or the René-Laennec clinic; 3) pregnant within 16 weeks of the first day of their last menses; 4) able to read and understand either French or English; and 5) provided written consent. Ethics approval was obtained from the CHU Sainte-Justine ethics committee.

The present study's design and data collection were previously described elsewhere[13]. However, for ease of understanding, the study methodology is summarized. At the end of their first prenatal visit, eligible women who accepted to participate were asked to complete a self-administered questionnaire at home. Data on demographic and socio-economic variables, NVP status and generic QOL were collected. Only women who reported suffering from NVP were asked to complete the NVPQOL questionnaire. This present validation study was done in the subgroup of pregnant women who reported suffering from NVP in the self-administered questionnaire.

### The NVPQOL

The NVPQOL questionnaire measures QOL in the last week and contains 30 items covering 4 general domains: physical symptoms and aggravating factors, fatigue, emotions, and limitations [12] (Table 1). Each item of the NVPQOL is measured using a Likert 7-point scale ranging from 1 (none of the time) to 7 (all of the time). The total NVPQOL score can be obtained by summing the 30 items (20^th ^item reversed) and ranges between 30 and 210. Lower scores correspond to better QOL.

**Table 1:** Domains and items of the NVPQOL questionnaire.

**Domains**	**30-Items**
Physical symptoms and aggravating factors	Nausea Sick to your stomach Vomiting Dry-heaves Poor appetite Symptoms worse in evening Not eaten for longer than you would like Worse when exposed to certain smells Worse when exposed to certain foods

Fatigue	Fatigue Worn-out, lack of energy Exhausted Tired

Emotions	Emotional Less interested in sex Downhearted, blue, sad, unhappy, depressed, gloomy Frustrated Fed up with being sick Reassured that your symptoms are part of normal pregnancy Can't enjoy your pregnancy

Limitations	Everything is an effort Accomplished less than you would like Took longer to get things done than usual Difficult or took extra effort to perform, and/or limited in types of work and other activities Difficulty maintaining your normal social activities with family, friends, neighbours, or social groups Rely on your partner to do things that you would normally do for family Difficulty looking after home Difficulty shopping for food Difficulty preparing or cooking meals Cut down on amount of time you spent at work or other activities

Source: Magee et al. 2002, with permission.

The NVPQOL has been developed using established procedures for the development of health-related QOL questionnaires that have been used for a wide array of medical conditions [12]. In addition, evidence of the content validity of the NVPQOL is available [12]. Content validity can be defined as the degree to which the sampling of items in the questionnaire reflects the concept being measured [14]. In fact, four sources of information have been used to generate the NVPQOL items in the initial development of the questionnaire: focus groups of women suffering from NVP, MEDLINE search, judgment from experts and clinicians, and a review of other validated health questionnaires. Moreover, factor analysis statistical procedures where use for the development of the NVPQOL, which also provides additional evidence to the content validity of the index [12].

### Generic quality of life

For comparison purposes, the generic health-related QOL was measured with the standard version (past 4 weeks) 12-item Short-Form Health Survey v.1 (SF-12) [15]. This shorter version of the commonly used SF-36 yields two summary measures: the physical component summary scale (PCS) and the mental component summary scale (MCS)[16]. Summary measures range from 0 to 100 and are calculated using the scores of the twelve items; higher scores represent better QOL. PCS and MCS scores were calculated with standard (United States) scoring algorithms and normalised using the US general population (mean, 50; SD, 10). The psychometric properties of the SF-12 questionnaire have been extensively evaluated in many different populations [15,17-19]. In fact, the SF-12 is highly reliable (test-retest correlations PCS = 0.86–0.89; MCS = 0.76–0.77)[15,20] and has shown a very good criterion validity as compared to the gold standard of health-related QOL, the SF-36 (PCS = 0.90–0.96; MCS = 0.93–0.97) [15,19].

### NVP severity

The severity of NVP was measured by the modified Pregnancy-Unique Quantification of Emesis and Nausea (modified-PUQE) [13]. This index is validated and measures NVP severity during a pregnancy's first trimester. The modified-PUQE is based on 3 physical symptoms of NVP: the extent of nausea in hours, the quantity of retching episodes, and the number of vomiting episodes on an average day since the beginning of the pregnancy. Total scores range between 3 and 15. When validated, the modified-PUQE was significantly associated with an outcome of direct importance for women who experience NVP such as QOL (SF-12 PCS: p < 0.0001; SF-12 MCS: p = 0.0008). Moreover, a substantial concordance was found between the modified-PUQE and the frequently used Motherisk PUQE. (ICC = 0.71) [13].

### Statistical analysis

Descriptive statistics were used to estimate the distribution of maternal characteristics and health-related QOL scores in the study population. Internal consistency, defined as the intercorrelations among items of a scale [21], was measured using Cronbach's alpha coefficients (α) for the complete NVPQOL questionnaire, and for each distinct domain. Cronbach's coefficients range between 0 (weak reliability) and 1 (perfect reliability). We considered the 0.7 cut-off as indicating acceptable internal consistency for research purposes. An α ≥ 0.9 shows excellent internal consistency and high reliability [14,21]. Secondly, criterion validity can be assessed by the extent to which a measure is able to predict the results of a gold standard [14]. Therefore, NVPQOL was compared to the SF-12. Linear regression models were built to measure the association between the NVPQOL score and the two SF-12 summary measures. Finally, linear regression models were built to measure the association between the NVPQOL score and the severity of NVP, a clinically relevant outcome. Significance was assumed at p < 0.05. All statistical analyses were performed using SAS Version 9.1 (SAS Institute, NC, USA).

## Results

A total of 367 pregnant women met all inclusion criteria, out of which, 288 reported NVP during the 1^st ^trimester (78.5%). Those who reported NVP in the 1^st ^trimester formed the study population.

Maternal characteristics including demographics, NVP severity, and health related QOL are presented in Table 2. The mean age of the participants was 32 (SD: 4.6) years while the mean gestational age at recruitment was 11 (SD: 1.8) weeks. Eighty-two percent of participants were Caucasian. As for QOL, the mean NVPQOL score was 94.8 (SD: 39.8). The mean physical component score on the SF-12 was 42.8 (SD: 9.1); the mean mental component score on the SF-12 was 45.9 (SD: 8.4).

**Table 2:** Demographics of women suffering from NVP.

**Characteristics (n = 288)**	**Measure**	
**Maternal age – yr **(mean ± SD)	31.65	± 4.62

**Gestational age – wk **(mean ± SD)	11.00	± 1.75

**Country of birth **– n (%)		
Canada	189	(65.63)
Other	99	(34.38)

**Race **– n (%)		
Caucasian	237	(82.29)
Asian	9	(3.13)
Black	26	(9.03)
Hispanic	16	(5.56)

**Rx insurance plan **– n (%)		
Provincial plan (RAMQ) only	81	(28.32)
Other insurance	205	(71.68)

**Work status **– n (%)		
Student or not working	72	(25.09)
Working	215	(74.91)

**Living arrangement **– n (%)		
With spouse or with someone (family or cotenant)	281	(97.91)
Living alone	6	(2.09)

**Education level **– n (%)		
University completed	185	(64.46)
University not completed	102	(35.54)

**Household income – CDN$/yr **n (%)		
Less than 40 000$	88	(31.43)
Between 40 000 et 79 999$	70	(25.00)
80 000 and over $	122	(43.57)

Comorbidities before pregnancy * – n (%)		
0	207	(71.88)
1	70	(24.31)
2 or 3	11	(3.82)

**Gravidity **– n (%)		
Multigravida	240	(83.33)
Primigravida	48	(16.67)

**Pre-pregnancy BMI **– n (%)		
Underweight or normal (BMI <25 kg/m^2^)	193	(68.68)
Overweight (25≤ BMI <30 kg/m^2^)	65	(23.13)
Obese (BMI ≥30 kg/m^2^)	23	(8.19)

**NVP severity **(mean ± SD)		
PUQE score	6.69	± 2.30

**NVP-specific QOL **(mean ± SD)		
NVPQOL score	94.82	± 39.84

**Physical and mental QOL **(mean ± SD)		
PCS SF-12 score	42.84	± 9.07
MCS SF-12 score	45.88	± 8.38

* Including asthma, anemia, depression, hypothyroidism, diabetes, epilepsy, hypertension and various problems like infections, eczema, migraines, etc.

### Reliability of the NVPQOL

Reliability of the NVPQOL was supported via internal consistency with Cronbach's alpha being larger than 0.8 for the whole questionnaire (α = 0.98) and for the 4 distinct domains respectively (Table 3).

**Table 3:** Cronbach's alphas for the NVPQOL questionnaire.

**Scale**	α
Whole questionnaire	0.98
Physical symptoms and aggravation factors domain	0.90
Fatigue domain	0.94
Emotion domain	0.86
Limitations domain	0.97

### Validity of the NVPQOL

**Figure 1:**
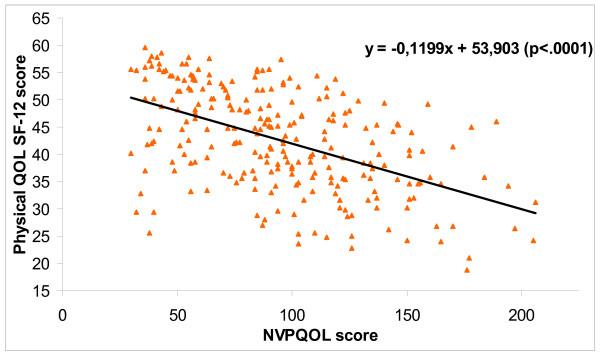
Association between NVPQOL score and physical QOL (SF-12, PCS).

**Figure 2:**
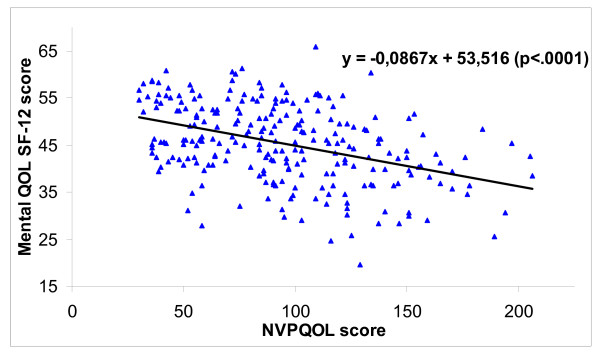
Association between NVPQOL score and mental QOL (SF-12, MCS).

When comparing NVPQOL and SF-12 QOL scores, lower NVP-specific QOL (higher NVPQOL score) was significantly associated with a lower QOL as measured by the SF-12 (PCS and MCS p < 0.0001; Figure 1 and 2)

### NVPQOL and NVP severity

When the NVPQOL and NVP severity scores were compared, NVP-specific QOL was significantly associated with more severe NVP symptoms as measured by the modified-PUQE (p < 0.0001).

## Discussion

Our study established internal consistency and criterion validity of the only existing NVP-specific QOL questionnaire. Reliability of the NVPQOL was supported by internal consistency for the complete questionnaire and the 4 distinct domains, respectively. We found a significant association between the NVPQOL score and the SF-12 physical and mental scores. The NVPQOL score was also correlated with severity of NVP symptoms.

Almost all estimated coefficients of internal consistency for the NVPQOL showed excellent reliability. In spite of the fact that the coefficient for the emotion domain was not above the 0.9 threshold, it showed acceptable internal consistency [14,21]. Although the test-retest reliability of the NVPQOL could not be measured within our study, the higher the internal consistency, the higher will be the test-retest reliability in theory [14]. As stated earlier, content validity of the NVPQOL has already been established [14]. The fact that this study showed an association between the NVPQOL and SF-12 scores is an argument towards its criterion validity since the SF-12 is a gold standard in terms of health-related QOL. Furthermore, the NVPQOL score correlated well with the NVP severity, which enhances the face validity of the NVPQOL given that QOL was found to be significantly associated with the NVP severity in the literature [11].

Generic QOL questionnaires are often used in pregnancy studies [22]. However, generic measures can be unresponsive to changes in specific areas of health [23]. The only validated pregnancy-related specific QOL questionnaire is the Mother-Generated Index [24], which assesses a mother's postnatal quality of life. As of now, no pregnancy-specific questionnaire allows for the evaluation of QOL during the gestational period. Because NVP is a prevalent condition in pregnancy, the NVPQOL questionnaire could be useful to perinatal epidemiological research. The use of the NVPQOL is justified since specific measures of QOL can enhance the detection of small, clinically important aspects in QOL related to specific areas of interest [12,23]. In fact, women suffering from NVP are likely to have distinctive concerns such as fears about antiemetic medication use during pregnancy [12]. Moreover, health-related QOL can be a good measure reflecting service needs and thus, it is useful to inform physicians [25]. For this reason, an NVP-specific index such as the NVPQOL could be a very useful tool in clinical practice in order to provide optimal management for women in need.

In our study, the time windows for which the generic and specific health-related QOL were measured were comparable. The standard version of the SF-12 covered the past 4 weeks and the NVPQOL covered the past week. Given that most of the NVP symptoms disappear by the 20^th ^week of gestation [2], we have good reasons to believe that the NVPQOL questionnaire could also be used for the second trimester of pregnancy, starting at the 15^th ^week of gestation.

The NVPQOL questionnaire has been previously reported to be suitable for all women with mild to severe NVP as well as having a good external validity [12]. Our study population is comparable to the Montreal population of pregnant women. The majority of women in our study cohort were Caucasians, which consequently improves the external validity of our results to Canadian population. Indeed, in 2001 less than 15% of the Canadian population belonged to a visible minority group [26].

## Conclusion

In conclusion, we established the reliability and validity of the only existing NVP-specific QOL questionnaire in a cohort of 288 pregnant women reporting NVP in the first trimester. Our results confirm that the NVPQOL is a reliable and valid index to measure NVP-specific QOL and that it is well suited for use during the first trimester of pregnancy. Globally, the NVPQOL can be a very useful tool in health research and clinical practice.

## Competing interests

The authors declare that they have no competing interests.

## Authors' contributions

Each author has participated actively and sufficiently in this study, and fulfils all authorship criteria of the International Committee of Medical Journal Editors. AL made substantial contribution to acquisition of data, analysis and interpretation of data, and drafting of the article. AB made substantial contribution to conception and design of the study, analysis and interpretation of data, and drafting of the article. Each author revised critically the manuscript and provided final approval of the version to be published.
